# Intrinsic disorder and conformational coexistence in auxin coreceptors

**DOI:** 10.1073/pnas.2221286120

**Published:** 2023-09-27

**Authors:** Sigurd Ramans-Harborough, Arnout P. Kalverda, Iain W. Manfield, Gary S. Thompson, Martin Kieffer, Veselina Uzunova, Mussa Quareshy, Justyna M. Prusinska, Suruchi Roychoudhry, Ken-ichiro Hayashi, Richard Napier, Charo del Genio, Stefan Kepinski

**Affiliations:** ^a^School of Biology, Faculty of Biological Sciences, University of Leeds, Leeds LS2 9JT, United Kingdom; ^b^Astbury Centre for Structural Molecular Biology, Faculty of Biological Sciences, University of Leeds, Leeds LS2 9JT, United Kingdom; ^c^Wellcome Biological Nuclear Magnetic Resonance Facility, Division of Natural Sciences, University of Kent, Canterbury CT2 7NJ, United Kingdom; ^d^School of Life Sciences, University of Warwick, Coventry CV4 7AL, United Kingdom; ^e^Department of Bioscience, Okayama University of Science, Okayama 700-0005, Japan; ^f^Centre for Fluid and Complex Systems, Coventry University, Coventry CV1 5FB, United Kingdom

**Keywords:** auxin, disorder, Aux/IAA, TIR1, IDP

## Abstract

AUXIN/INDOLE 3-ACETIC ACID (Aux/IAA) transcriptional repressor proteins and the TRANSPORT INHIBITOR RESISTANT 1/AUXIN SIGNALING F-BOX (TIR1/AFB) proteins to which they bind act as auxin coreceptors. While the structure of TIR1 has been solved, structural characterization of the regions of the Aux/IAA protein responsible for auxin perception has been complicated by their predicted disorder. Here, we use NMR, CD and molecular dynamics simulation to investigate the N-terminal domains of the Aux/IAA protein IAA17/AXR3. We show that despite the conformational flexibility of the region, a critical W–P bond in the core of the Aux/IAA degron motif occurs at a strikingly high (1:1) ratio of *cis* to *trans* isomers, consistent with the requirement of the *cis* conformer for the formation of the fully-docked receptor complex. We show that the N-terminal half of AXR3 is a mixture of multiple transiently structured conformations with a propensity for two predominant and distinct conformational subpopulations within the overall ensemble. These two states were modeled together with the C-terminal PB1 domain to provide the first complete simulation of an Aux/IAA. Using MD to recreate the assembly of each complex in the presence of auxin, both structural arrangements were shown to engage with the TIR1 receptor, and contact maps from the simulations match closely observations of NMR signal-decreases. Together, our results and approach provide a platform for exploring the functional significance of variation in the Aux/IAA coreceptor family and for understanding the role of intrinsic disorder in auxin signal transduction and other signaling systems.

## Significance

This paper shows the most detailed and complete view to date of a canonical Aux/IAA auxin coreceptor protein. Molecular dynamics simulation, coupled with nuclear magnetic resonance analysis shows that, although nominally disordered, the N-terminal half of the Aux/IAA AXR3 appears to show a propensity toward adoption of a small number of specific conformations. The conformational coexistence in auxin coreceptors provides an insight into a protein family that is so crucial for plant life on earth.

Auxin is a central signaling molecule in plant biology, with a fundamental role in developmental events and in the regulation of cellular growth. This capacity for control arises from its ability to directly alter gene expression levels, as well as from indirect mechanisms (1–9). The first step for all such cascades of molecular events is the binding of auxin (indole-3-acetic acid, IAA) to a member of its receptor family, whose canonical representative is Transport Inhibitor Response 1 (TIR1) (10, 11). Subsequently, transcription-regulating proteins known as the Aux/IAAs bind on the TIR1–auxin system, completing a TIR1–ubiquitin E3 ligase complex on which the Aux/IAA coreceptors are ubiquitylated, leading to their degradation and starting the derepression of gene expression (6, 12, 13). Thus, auxin acts as a molecular glue whose presence is necessary for the assembly of the final complex. The mechanisms of selectivity of auxin binding have been investigated, highlighting the importance of the biochemical properties of the residues that form the deep binding pocket of TIR1 (14). However, little is known yet about the processes determining the association of the Aux/IAA coreceptors with the initial complex. The only existing results have established the importance of a degron sequence in domain II of all Aux/IAAs (10, 11, 15), as well as the role of the residues immediately surrounding it in guiding the assembly of the final complex (16). Other studies have highlighted that while the degron motif is necessary for binding, nearly the whole N-terminal half of the protein is needed for full activity (17). This indicates that the interaction between TIR1 and Aux/IAA is complex and goes beyond the recognition of the core degron sequence motif. In turn, this suggests that diversity in regulation between different members of the Aux/IAA family may be caused by sequence variation outside the degron motif, calling attention to the relevance for the process of the entire Aux/IAA proteins. Thus, to characterize the formation of the coreceptor complex, it is important to establish the structural characteristics of a full-length Aux/IAA protein and determine how these relate to the interactions mediated by the degron.

The highly conserved C-terminal Phox and Bem1p (PB1) domain of the Aux/IAA17/AXR3 protein is solved (18, 19), but knowledge of the N-terminal half is fragmentary because its structure is believed to be disordered (20). One study has shown that an Aux/IAA monomer can form contacts with the flanks of TIR1 and suggested that the domain where the degron is located can loop across the surface of the receptor (16). However, the structures assumed by the N-terminal domains and the interactions of the full protein with TIR1 remain largely unexplored.

Here, we combine NMR and CD (circular dichroism) analysis with molecular dynamics (MD) simulations to study the disordered amino-terminal of the Aux/IAA protein AXR3 and to estimate its interactions with TIR1. Our results show that the degron harbors an exceptionally abundant *cis* proline at its heart, within an N-terminal featuring two main arrangements of folded elements that exist possibly in a dynamic equilibrium with each other and with further, more disordered, states. Combining these with the previously reported structure of the C-terminal domain, and performing extensive simulations, we estimate a weighted map of contacts between AXR3 and the TIR1–auxin complex, which is supported by NMR signal-decrease experiments. Our study suggests three key points. First, the Aux/IAAs belong to a class of intrinsically disordered proteins (IDPs) characterized by conformational alternatives that may contribute to their interactions. Second, folded elements can feature in the ensemble of conformations, resulting in more structured Aux/IAAs than first thought. Finally, the two main conformational arrangements of the N-terminal half form different auxin coreceptor complexes, which may display functional differences.

## Results

### The N Terminus of AXR3 Supports a Very High Abundance of the *Cis* Conformation of the Key Degron Residue Proline 87.

To investigate the structural biology of the unfolded N-terminal domains of AXR3 we used NMR. For these experiments, we excluded the characterized C-terminal PB1 domain to focus on the regions of the protein directly involved in auxin perception and avoid multimerization that would otherwise mask elements of the analysis. The N-terminal half of AXR3 was expressed as a ^13^C, ^15^N isotopically labeled protein. A combination of amide proton and carbon-detected NMR backbone assignment experiments recorded at 950 MHz led to an essentially complete backbone assignment. The following experiments were used: HNCA, HNcoCA, HNcaCB, CBCAcoNH, HncaCO, HNCO, CON, hCACO, and hCAnCO (overview: https://protein-nmr.org.uk/solution-nmr).

**Fig. 1.**
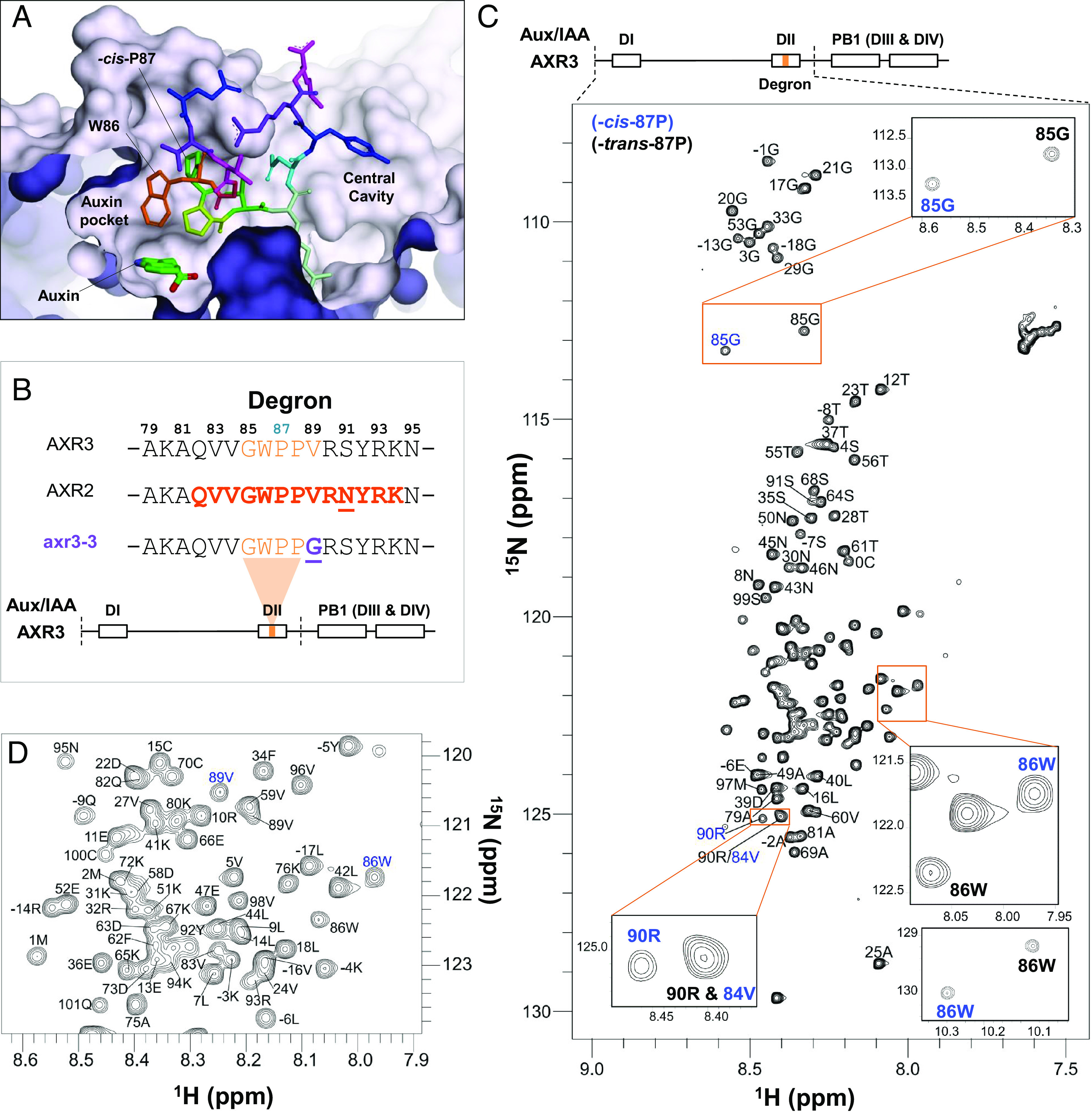
Overview of the Aux/IAA degron and the intrinsic disorder of AXR3 DI/DII. (*A*) Structure of IAA7/AXR2 degron (*cis*-P87) bound to TIR1 and auxin, showing the two TIR1 cavities based on 2P1Q ((12)). The molecular surface of TIR1 is shown in mauve, the degron peptide in colored sticks by residue and auxin is green at the base of the auxin-binding pocket (*B*) Amino acid sequences of DII from different Aux/IAA proteins with polymorphisms highlighted in bold and underlined. Core residues are in orange, and the mutated residue in axr3-3 is shown in purple. The AXR2 sequence highlighted and in bold indicates the peptide crystallized by Tan et al. (12). Below the sequence alignment is a schematic of the AXR3 protein showing the four domains. The location of the degron is highlighted, and the dashed lines indicate the DI/DII region of the protein studied by NMR (*C* and *D*) ^1^H–^15^N HSQC spectrum of the protein AXR3 DI/DII at 16.5 °C. The peaks associated with P87 in the *cis* isomer conformation are annotated light-blue. (*D*) An enlarged image of the signal-dense region of the HSQC spectrum in (*C*).

The NMR data show an extensive region of intrinsic disorder encompassing the majority of the N-terminal domain (Fig. 1). The ^1^H–^15^N heteronuclear single quantum correlation (HSQC) spectrum for AXR3_1-101_ is characterized by signals occurring in a narrow ^1^H chemical shift region (7.9 to 8.6 ppm), indicative of an IDP (Fig. 1 and *SI Appendix*, Table S1).

**Fig. 2.**
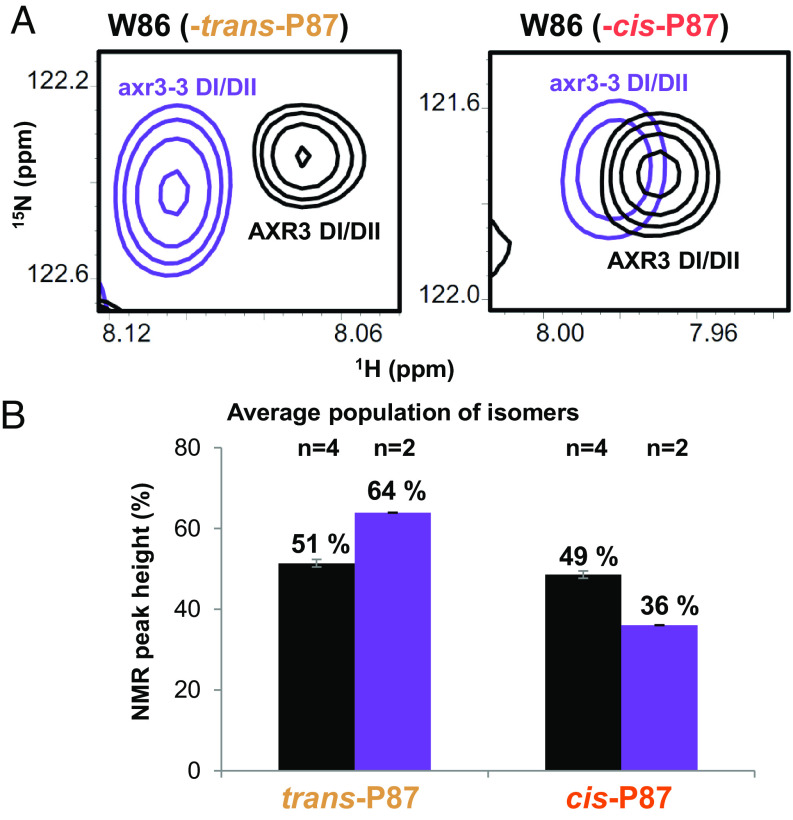
Proline 87 within the degron core of AXR3 exhibits a high *cis*:*trans* ratio. (*A*) HN cross peaks associated with W86 in AXR3 (black) compared to those observed for the axr3-3 mutant (purple). (*B*) Average *cis* and *trans* isomer ratios determined from the HN cross peak heights recorded for the G85 and W86 signals in AXR3 and axr3-3, black and purple, respectively. The number above the bars indicates the number of repeats; the error bars are the SD of the individual experiments.

During our NMR analysis of the N-terminal half of AXR3, the results showed a clear splitting of resonances for residues in the degron, particularly for G85 and W86, for which both *cis-* and *trans*-linked states were visible, with no overlap from neighboring peaks in the spectra (Fig. 2*A*). Two states represented by peaks of approximately equal intensity could be identified for all residues from V83 to S91. For all these, two continuous separate backbone assignment paths were constructed to confirm the separate assignments of the *cis* and *trans* states and identify the *cis*/*trans* proline equilibrium of P87 as the origin for the two states. This was greatly helped by the availability of 950 MHz carbon-detected data. The isomer ratio determined from the height of the HN cross peaks was found to be 49:51, *cis*:*trans* at 16.5 °C (Fig. 2), a temperature consistent with plants growing in a temperate climate. This represents an unusually high proportion of the *cis* state even for a proline preceded by an aromatic residue, a combination which has been found to display an elevated population of the *cis* state of between 20 and 35% (21). Importantly, it is the *cis* isomer that is observed by crystallography in the bound complex (12; Fig. 1*A*).

To assess the sensitivity of the isomerization state of P87 with respect to adjacent residues, we performed ^1^H–^15^N HSQC analyses on a variant of AXR3 in which V89 was substituted to glycine. This change represents the causal mutation in the well-characterized auxin-resistant mutant *axr3-3* (22, 23). HSQC analysis of the N-terminal half of the axr3-3 protein revealed a *cis*:*trans* ratio for P87 of approximately 1:2, a notable decrease in the occurrence of the *cis*-P87 state relative to the wild-type protein (Fig. 2*B*). This indicates that the conserved valine contributes to the stability of the W86–P87 *cis* isomer, emphasizing the importance of the conserved degron motif.

### Generating an Initial Model for the N-Terminal Domains of AXR3.

The unusually high population of *cis*-P87 (24) hints at elements or patterns of structure not revealed by NMR analysis. To delve further into the structural biology of the N-terminal domains of AXR3 we took a computational approach. In principle, good models of proteins whose structure is not known can be obtained via comparative modeling. In the case of the N-terminal half of AXR3, there are no suitable templates to inform homology modeling, and so, we used DMPfold (25) to verify whether machine-learning methods could provide a structural prediction with high confidence. This did not occur, with the method only providing an effectively unfolded structure, but, notably, with the W86–P87 bond inside the degron in *cis* conformation, consistent with our NMR analysis (Fig. 2*A*) and with the structure of the degron solved in complex with TIR1 and auxin (12). These two residues are located centrally within the bound degron, and a *cis* bond appears to be necessary to allow pi-stacking to occur between them and the molecule of auxin within the TIR1 binding pocket.

### Evidence for Structure within the N-Terminal Domains of AXR3.

Since the DMPfold prediction is an unfolded, physically plausible structure, without steric clashes, we decided to use it as starting point for an extensive conformational search using MD simulations, to explore the landscape of configurations available to AXR3. Traditional in silico methods are known to be challenged by IDPs due to the low-energy barriers between neighboring states and the complexity of the configuration space (26–29). However, the development of interaction-based replica-exchange methods has been shown to allow for extensive sampling of IDP conformations (30). The method is particularly useful for large proteins that are not amenable to other accelerated techniques because of the numerical instabilities they would cause. Our approach specifically combines the replica method with self-guided Langevin dynamics (RXSGLD) (31).

**Fig. 3.**
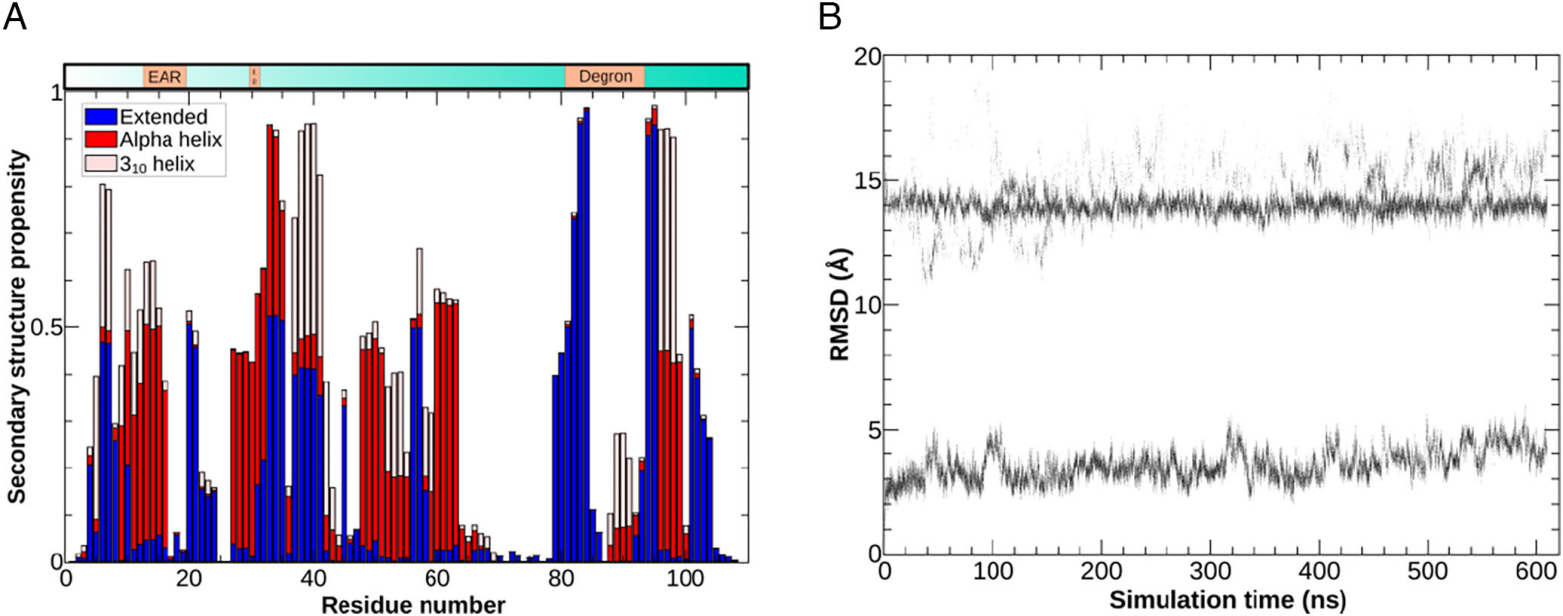
In-silico evidence of disorder in the N-terminal domain of AXR3. (*A*) Secondary structure propensities of the N-terminal half of AXR3. Only 12% of the regions have a propensity greater than 0.8 for displaying secondary structure and most of these switch between alternatives, while 35% have a propensity greater than 0.8 to be in a random coil state. The sequences defined as “extended” can be interpreted as beta strands. (*B*) RMSD of the atomic positions in each frame, with respect to their initial ones, showing a separation of the data into multiple bands, indicative of multiple, different, folded structures.

Estimating the secondary structure propensity from the RXSGLD trajectory reveals a complex picture (Fig. 3*A* and *SI Appendix*, Figs. S2–S4). First, 43% of the N-terminal half of AXR3 has less than 20% propensity of assuming any specific folded secondary structure at any time. Indeed, for more than 80% of the time, 35% of the residues are in random coils. Also, most areas of the sequence showing a high propensity for any fold type are around 3 residues in length, consistent with low overall order in the protein. The exception is a run of residues adjacent to the ethylene-responsive element binding factor-associated amphiphilic repression (EAR) domain that shows up as a turn of alpha helix.

To further characterize the behavior of these domains, we measured the scaling properties of the gyration radius of increasingly longer segments of the protein. In disordered proteins that behave like pure random coils or self-avoiding polymers, this quantity follows a power law with an exponent between 0.5 and 0.588 (a behavior commonly known as Flory scaling) (32–36). However, this appears not to be the case for AXR3 (*SI Appendix*, Fig. S5), raising questions about the nature of disorder in this protein. We next measured the ratio of the average gyration radius to the rms end-to-end distance over the trajectory of the simulated protein, finding this to be approximately 0.449. Again, this value agrees with the lack of Flory scaling, since the expected ratios from this calculation for random coils and self-avoiding polymers are 0.408 and 0.406, respectively. However, values close to our result for AXR3 have been observed for other “anomalous” IDPs (37). The behavior of these proteins is explained by the coexistence of sizeable populations of different structured conformations. Thus, we hypothesized that a similar behavior may be found also in AXR3. Our assumption is supported by the banding of the RMSD plot (Fig. 3*B*). Normally, if one computes the RMSD of the frames of an equilibrated MD trajectory with respect to the first one, one expects to find low values with a slow increase over time. This is clearly visible in the lower band of the plot, starting at approximately 2 Å. However, here, we also note the presence of a different band of RMSD values, which stays flat at about 14 Å. Since the RMSD is computed after fitting each frame to the reference, this indicates the presence of a substantially different structure within the trajectory, which is sampled for a time comparable to the initial one.

To cluster the different structures, we introduced the *structurally weighted RMSD* (SWRMSD; see *Methods*), which yielded 12 different clusters. Of these, the two largest have almost the same occupancy and comprise approximately 91% of all frames, which suggests that they dominate the ensemble of structures occupied by the N-terminal half of AXR3. These results confirm our hypothesis that the N-terminal half of AXR3 exists as a population that includes different partially structured conformations.

**Fig. 4.**
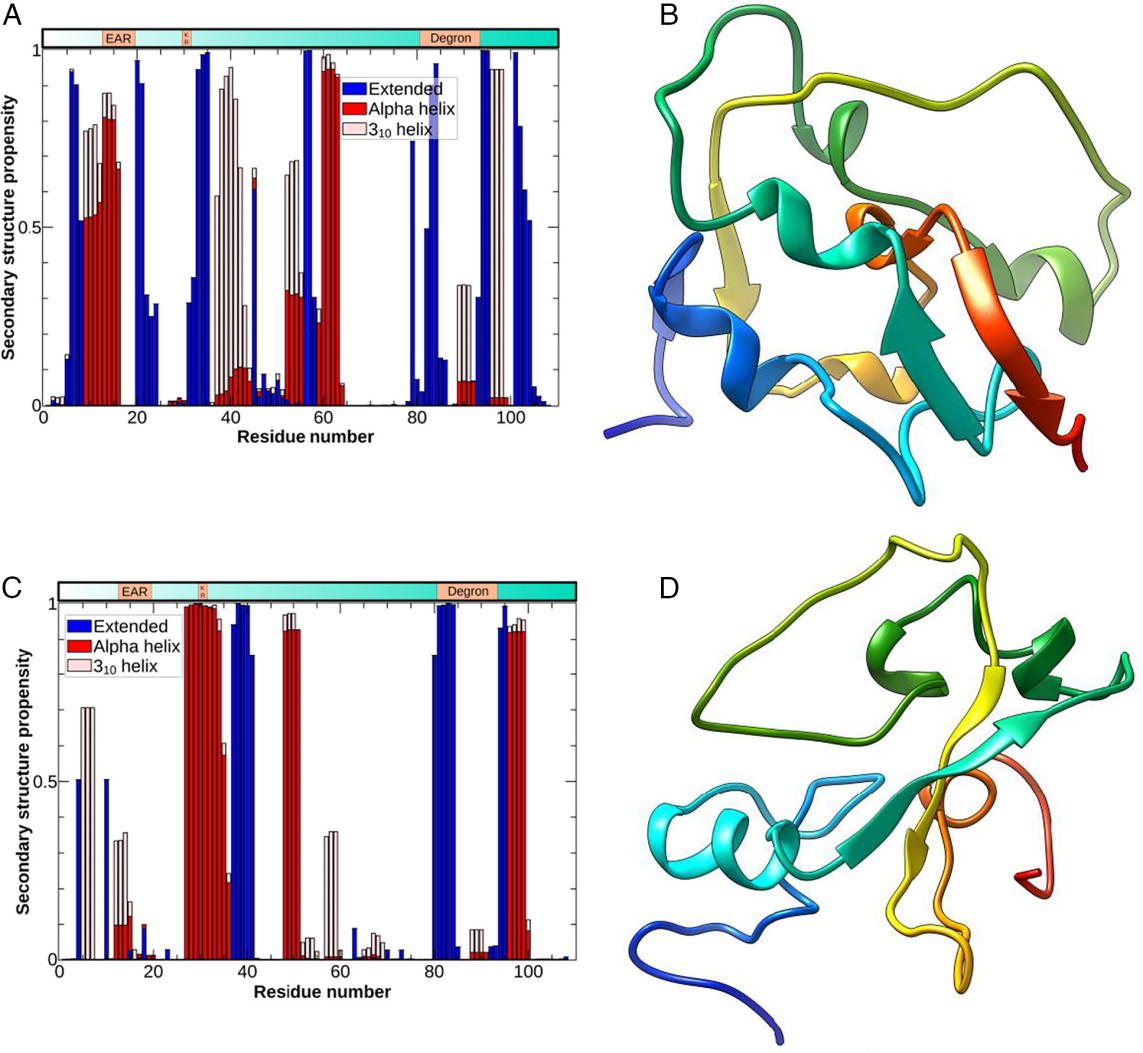
The N-terminal domain of AXR3 preferentially forms two distinct partially structured conformers within the ensemble: (*A*) and (*B*) cluster 1, (*C*) and (*D*)cluster 2. (*A*) and (*C*) Secondary structure propensities for the first and second cluster of the N-terminal half of AXR3, respectively. (*B*) and (*D*) Structures from representative frames of the first and second clusters of the N-terminal half of AXR3, respectively. The coloring, passing from blue at the N terminal to red at the C terminal, allows for an easy visual detection of the substantial differences in structural arrangement between the two clusters.

**Fig. 5.**
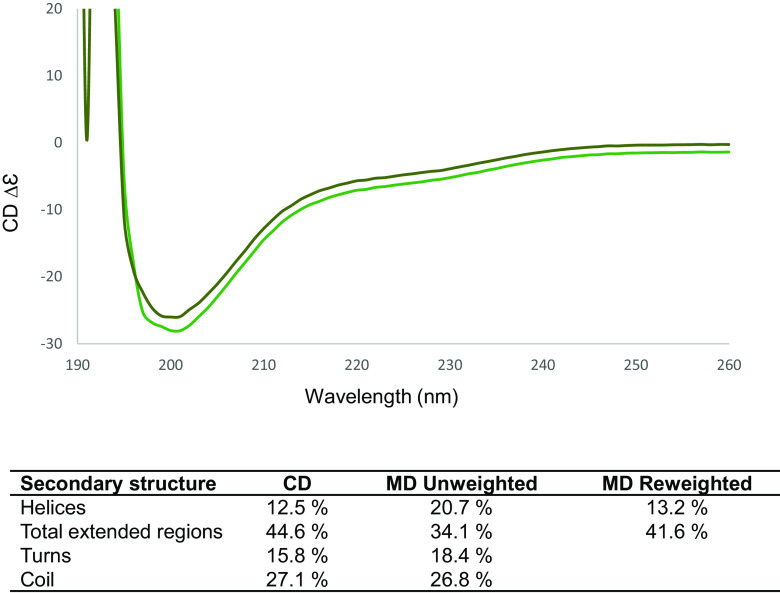
CD results are consistent with the secondary structure propensities observed in the MD ensemble. (*Top*) The CD spectrum of the N-terminal half of AXR3. Replicate datasets are shown by green lines. (Base) The table summarizes the secondary structure predictions for CD and MD. In the final column, the MD values for helices and extended regions have been reweighted to correct for the force fields used (39, 40).

The high secondary structure propensities within each cluster show clearly separated regions found always as either coil, helix, or beta strands (Fig. 4 *A* and *C* and *SI Appendix*, Figs. S7–S12), although the tertiary arrangement of folded regions is substantially different between clusters (Fig. 4 *B* and *D*). In fact, only the short stretch around residue 80 is likely to retain the same, beta-sheet structure between the two clusters. Thus, our initial molecular dynamics simulations have identified two dominant, but distinct, structural preferences for the N-terminal half of AXR3 within a structural ensemble. The structural assignments from the MD are consistent with data from CD spectroscopy of the N-terminal half of AXR3 (Fig. 5) especially when the data for the MD ensemble are reweighted to reflect the known force field bias (38, 39).

**Fig. 6.**
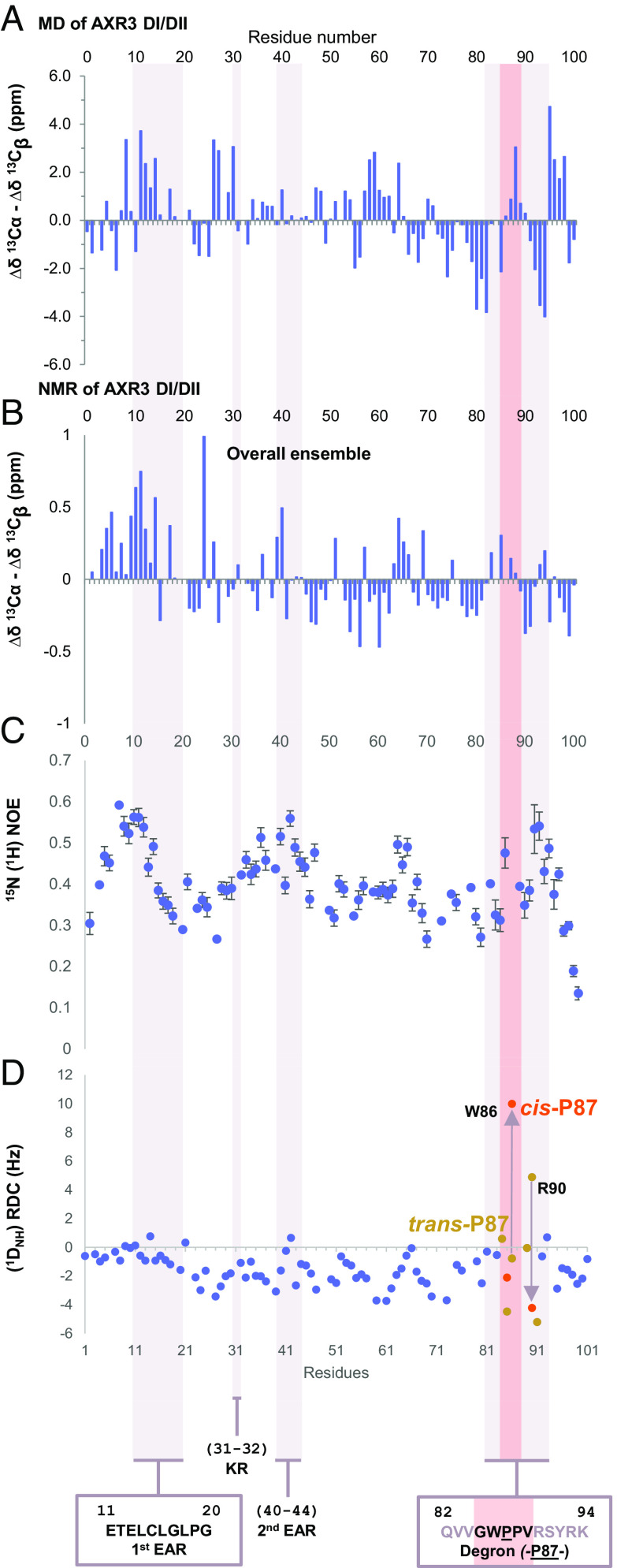
Experimental verification of the structure of the N-terminal domain of AXR3. (*A*) Chemical shift differentials computed from the MD trajectory. (*B*–*D*) An NMR structural study of AXR3 DI/DII, residues 1 to 101. The characterized sequence motifs of the Aux/IAA are highlighted by the shaded bars, shown on the graphs right-side of figure. An additional shaded bar in pink shows the region of the conserved degron core. Only the data for the degron core can be separated into *trans*-P87 or *cis*-P87 isomer states. The *trans*-P87 degron is shown, as it is the most complete NMR dataset. (*B*) The chemical shift differential (∆δ, ppm) between ^13^C_α_ and ^13^C_β_ signals assigned to residues along the carbon backbone of AXR3 DI/DII. Positive ∆δ indicate a tendency for helical secondary structure. Negative ∆δ indicate a tendency for β-secondary structure. (*C* and *D*) Peaks in the data points infer more structured regions within AXR3 DI/DII, measured on a 950-MHz spectrometer. (*C*) ^15^N (^1^H) heteronuclear NOE profile for AXR3 DI/DII. (*D*) Backbone amide (^1^D_NH_) RDC profile for AXR3 DI/DII. The effects of *cis-trans* isomerization, neighboring residues are color coded for the two isomer states as follows. The data points for the *trans*-P87 isomer state are shown in gold, and the *cis*-P87 conformation is shown in red. Arrows mark the change in data point position between the isomer states of the W–P bond. RDC data for W86 and R90 both show a clear change in conformational orientation for each isomer state.

NMR provides an overall view of the ensemble of conformations occupied by the protein during data collection and the presence of distinct conformers complicates structural interpretation of our NMR data. In order to make a comparison between MD and NMR, C_α_–C_β_ chemical shift differences (Δδ^13^C_α_–δ^13^C_β_) were computed from the MD trajectory and averaged over the complete production run of the molecular dynamics calculation with respect to the values expected for a random coil (Fig. 6*A*). The results showed a distinctive pattern of elements of structure within the disorder, notably around the EAR motif and the degron. This distinctive pattern in chemical shifts is also observed in the NMR ensemble data (Fig. 6*B*), albeit more dilute. The chemical shift data are also consistent with nuclear overhauser effect (NOE) and residual dipolar coupling (RDC, ^1^D_NH_) measurements, where peaks correspond to these same elements of structure (Fig. 6 *C* and *D*). Taken together, all the analyses support a model in which the N-terminal half of AXR3 exists as a set of partially structured conformations. This behavior is consistent with the results of the analysis of the AXR3 N-terminal according to the Das–Pappu model (40), which we performed using CIDER (41). These show that the sequence is predicted to be a “Janus sequence”, whose conformation can be extended or compact depending on the environment and on interactions with ligands or other proteins (*SI Appendix*, Fig. S13). To the best of our knowledge, no IDP in which the disorder is due to a glassy landscape (42) with multiple semifolded structures has been characterized, even though their existence has been hypothesized and their behavior partially explored (37).

### The Coreceptor Complex.

For the next step toward the goal of studying the interactions between AXR3 and TIR1, we created models of the full-length protein by attaching the known structure of its C-terminal half to each of the two principal structures of the N-terminal half and relaxed each resulting model. We then performed targeted MD simulations to drive each model toward the binding pocket of an already-relaxed TIR1/auxin complex. As guiding constraints, we used the relative positions of the auxin-binding pocket and the conserved GWPPVR motif of the degron, inferred from the crystal structure (12).

**Fig. 7.**
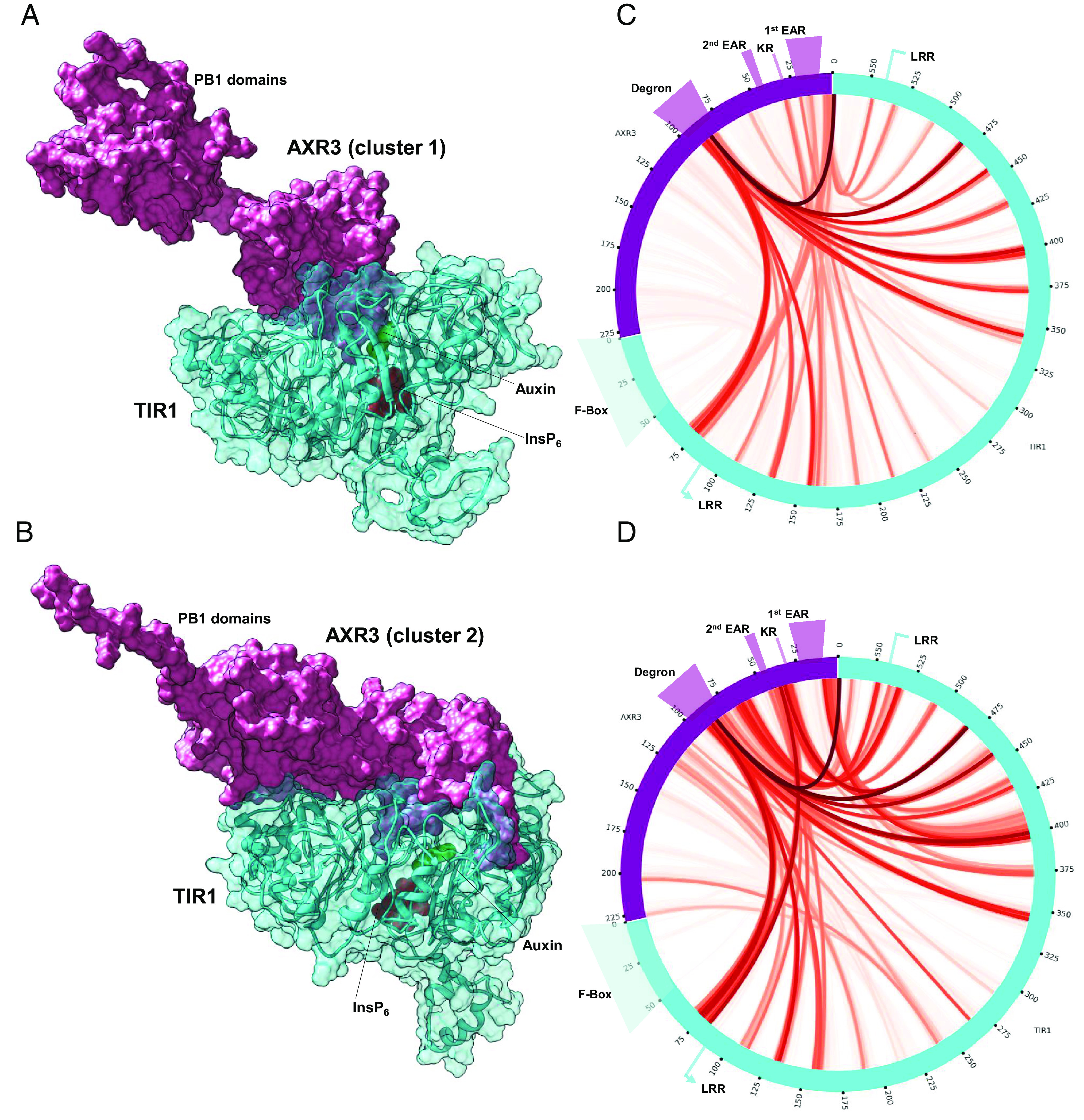
The two main conformational clusters of the AXR3 N-terminal half form different contacts with TIR1. Minimum-energy poses of the full AXR3-TIR1 models constructed using cluster 1 (*A*) and cluster 2 (*B*). AXR3 is in solid surface representation (pink); TIR1 is in semitransparent surface/ribbon representation (cyan); also shown are the molecular surfaces of auxin (green) and the structural cofactor inositol hexakisphosphate (InsP6). The images are orientated to focus on the different conformations of the PB1 domains in the bound state for the two main clusters. (*C*) and (*D*) native contacts between TIR1/auxin complex and cluster 1 and cluster 2, respectively. The color scale is such that the weakest nonzero contact is pale-red, and the strongest contact is dark red.

Since we are interested in determining the contacts between TIR1 and AXR3 in the bound state, we coarse-grained the models to allow for a long simulation run from which to gather good statistics. The results show that the two clusters form similar, but different sets of contacts with TIR1, adopting somewhat different orientations in the structure of the complex (Fig. 7 and Movies S1 and S2).

**Fig. 8.**
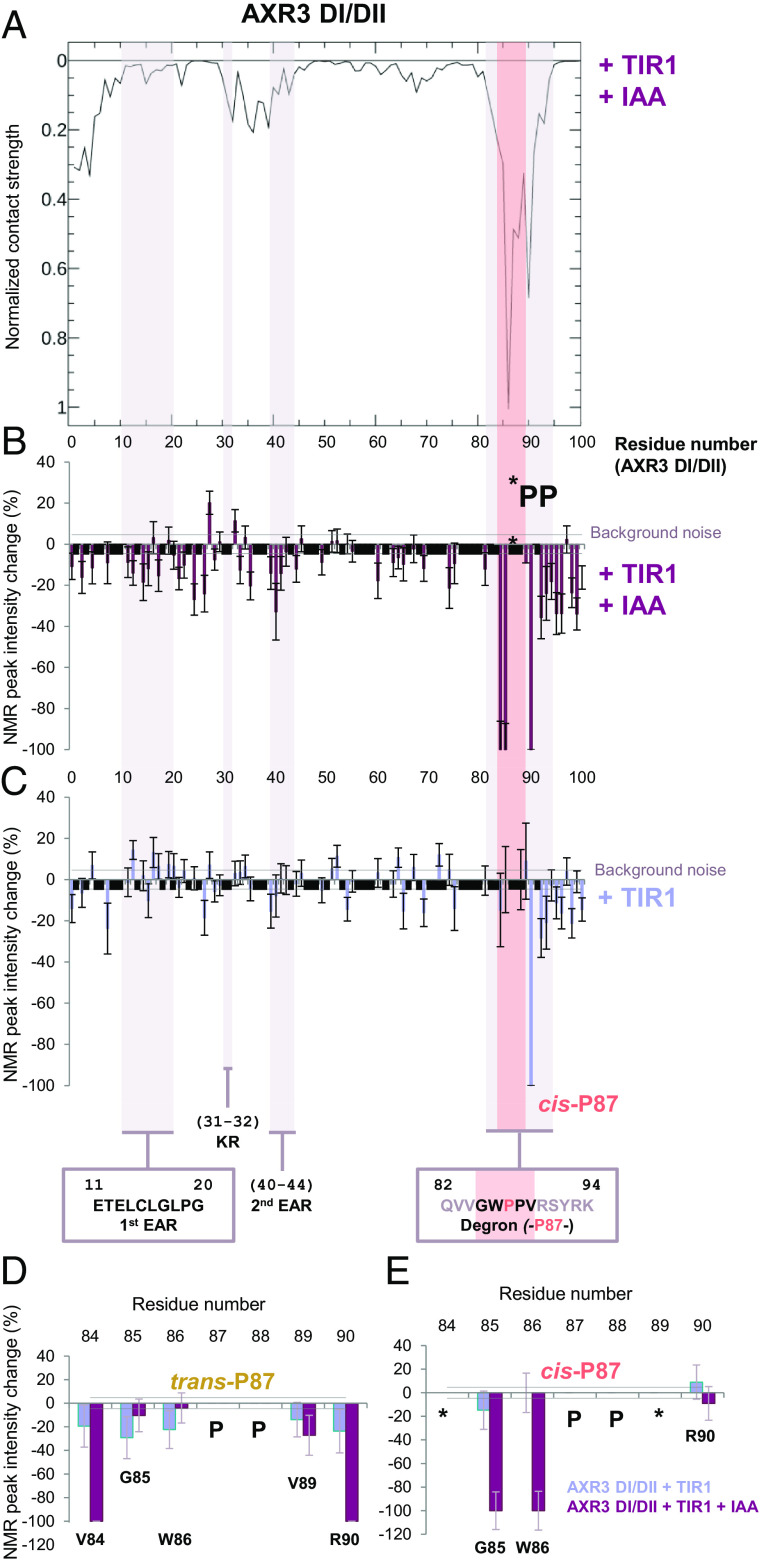
Contacts between AXR3 and the TIR1. (*A*) Overall strength of contacts between AXR3 and the TIR1/auxin complex from MD analysis, shown per residue of AXR3. The strengths are an average of those of the two main clusters, weighted by relative occupancy, and normalized to that of the strongest contacting residue, i.e., W86. The black boxes represent areas too weak to follow. (*A*–*C*) Important biological motifs are annotated and include the EAR motifs and the degron, these regions are shaded on the graphs. The pink shaded bar indicates the degron core. Data from the degron in the *cis*-P87 conformation is shown. (*B*–*E*) Percentage changes in the intensity of HN cross peaks from ^1^H–^15^N HSQC spectra of AXR3 DI/DII with the addition of TIR1 (blue), and TIR1 with IAA (purple). A change of −100% indicates that peak intensity has decreased to the noise floor and is no longer observed. The region V84 to R90 shows clear splitting of resonances associated with either *cis-* or *trans-*P87 degron conformers [percentage changes for the *trans-*P87 degron are shown in (*D*), and *cis-*P87 V84 to R90 in (*E*)]. The background noise in two forms, as error bars, and as a bar along the *x* axis of each graph. The error bars represent the background noise as a percentage of maximum peak intensity for each HN cross peak. The horizontal bar shows the average background noise in the spectrum as a percentage of maximum peak intensity. (*B*) and (*C*) Black-solid bars on the graph indicate residues for which peak intensity could not be measured due to HN peak overlap or where prolines are positioned in the sequence, the degron di-proline is indicated (PP). (*D*) and (*E*) The AXR3 degron in the *trans*-P87 and *cis*-P87 conformations, showing the percentage differences with the addition of TIR1 and IAA. Missing data points where the peak intensity could not be measured due to HN peak overlap are indicated with the symbol (*); prolines are indicated with the symbol (P).

To verify these predictions, we carried out NMR experiments (^1^H–^15^N HSQC), from which the observed changes in linewidths and peak heights allowed us to infer which parts of AXR3 make strong contacts with TIR1 (43, 44). Given the continually changing order in AXR3, regions in close contact with TIR1 will show signal intensity loss. In contrast, other regions of AXR3 will still exchange and show sharp peaks in the NMR data. The results are compared to the average values from our simulations obtained by weighing the contribution of each cluster in the ensemble (Fig. 8*A*). The two datasets are strikingly similar, showing that the experimentally measured loss of signal intensity validates the overall marginal distribution of simulated contacts. Decreases in NMR peak intensity from AXR3 associated with the addition of TIR1 in the presence and in the absence of auxin showed the degron as the predominant binding interface, supported by the adjacent C-terminal amino acids (Fig. 8 *B* and *C*). The G85 and W86 HN cross peaks associated with the *cis* isomer of P87 display some of the largest changes when auxin is present, with their NMR signals no longer observed. It is notable that in the *trans* state of P87, these two core residues only show limited engagement with TIR1 in the presence of auxin, whereas the more distal residues V84 and R90 showed large peak intensity losses (Fig. 8 *D* and *E*). This suggests that the *trans* conformer can engage in the presence of auxin but in a different pose and substantially outside the pocket.

Our NMR analysis also showed that the binding interface extends beyond the peptide used in crystallography, which ended at K94. In fact, we observed changes in signal intensity up to residue Q101, suggesting that the binding interface between the two proteins extends well past the core degron, a finding that is consistent with previous studies (16, 17, 45) and with our MD simulations (Fig. 7).

Overall, the data suggest a picture of a protein that shows multiple conformations at the level of secondary and tertiary structures. Importantly, NMR and MD datasets suggest these alternative conformers do not prevent complex formation with TIR1 and may indeed be necessary to maintain a high cis-conformer ratio ready for auxin action.

## Discussion

We have combined NMR analysis with molecular dynamics simulations to study the N-terminal domains of the Aux/IAA protein AXR3. The resulting MD models were combined with the previously reported solution structure of the C-terminal PB1 domain (PDB code 2MUK) (18) to form the first images of a full-length Aux/IAA protein (Fig. 7).

The feature of Aux/IAAs that is critical for auxin coreceptor assembly is the degron. In all the crystal structures of the bound complex, the degron assumes a W–*cis–*P imide bond. This is uncommon, as less than 5% of prolines are normally found in a *cis* isomer, even within disordered proteins (46). Our NMR data showed that in the unbound AXR3, the *cis:trans* isomer ratio is an exceptionally high 1:1 (Fig. 2*B*). The study of proline isomers is challenging because HN-based NMR methods do not register prolines and most computational methods are challenged by the energy barrier. Fortunately, we have been able to identify the presence of both isomeric forms in the protein by using the shift in HN cross peaks associated with the adjacent residues, notably W86, in the AXR3 degron, and the data are consistent with the results achieved using new MD force fields and methodologies such as unbiased replica exchange of self-guided Langevin dynamics simulations (31).

Studies on the mutant *axr3-3* protein showed that a change in the core degron sequence from WPPV to WPPG led to a reduced *cis:trans* ratio, indicating that the stability of the *cis* conformer is acutely sensitive to degron sequence (Fig. 2). Many other degron mutants, some with extreme phenotypes, have been reported, although the phenotypes have been explained so far in terms of sequence, not mechanism (47). The *axr3-3* mutant is hypersensitive to auxin (23) and this phenotype is consistent with a smaller pool of *cis* conformer. Some animal signaling systems make use of prolyl-*cis-trans* isomerases and, intriguingly, an Aux/IAA compatible *cis-trans* isomerase, LRT2, has been found in rice (47). However, no such isomerase has yet been identified in Arabidopsis and whilst the *trans* isomer can bind to TIR1, probably as an encounter complex, our data neither support nor dismiss the possibility that this complex can help to catalyze proline isomerization in the degron.

The MD simulations of full-length AXR3 protein have enabled us to estimate interactions in the canonical auxin coreceptor complex in addition to those involving the degron (Fig. 8*A* and *SI Appendix*, Figs. S13–S15). The simulations suggest that each structure assumed by the N terminal interacts with the auxin-bound TIR1 somewhat differently, creating distinctive contact plots. Other work has linked the N-terminal KR rate motif (K31-R32) and a touch-point in PB1 to complex association, perhaps helping to loop the Aux/IAA across the TIR1 surface for IAA7 (16). The KR and PB1 domains influence the rate of Aux/IAA degradation (22), and although they are not critical for auxin-mediated binding (10, 11, 15), our simulations identify the same contact areas for IAA7 and AXR3 (Fig. 7 *A* and *B*) (16), supporting the concept, that when bound, Aux/IAAs are extended across TIR1 to facilitate ubiquitylation.

The N-terminal part of the IAA7 protein was expected to lack order (16, 20), but we observed a structurally complex ensemble. The data suggest that, whilst nominally disordered, there appears to be a propensity toward adoption of a small number of specific designs. Importantly, the two main conformational subpopulations of the N-terminal half appear to influence the deployment of the PB1 domains. Such differences would be expected to influence the biological interactions and hence function and consequently, open up new avenues for investigation. Furthermore, it is not yet possible to explain the high incidence of *cis* conformer in the degron in terms of the disorder or the small elements of structure in the IDP. However, given that the N-terminal half of all Aux/IAAs are IDPs, it is possible that this balance between order and disorder contributes to the propensity for *cis*-proline in the degron. It will be important to explore the context of Aux/IAA degrons further whether we are to understand this feature that is essential for plant life on Earth.

## Methods

### Molecular Dynamics Simulations.

All MD simulations were carried out with the AMBER suite (48). To prepare an initial system for the RXSGLD, we started from the DMPFold guess for the structure of the N-terminal half of AXR3, using the pdb4amber and reduce tools to add hydrogen atoms and obtain a pdb output (49). We then produced parameters and topology files with LEaP, choosing to use the ff14SBonlysc force field, which combines the backbone parameters of the ff99SB force field (50) with the side chains ones from the ff14SB force field (51). This was motivated by our intention to perform this part of the simulations in implicit solvent, using the generalized Born model in the formulation of ref. 52 with optimized atomic parameters (53), an approach that has shown to yield the most realistic results when combined with the specific set of protein parameters as obtained via this particular choice of force field combination (54). After the preparation of parameters and topology, we minimized the system via the steepest descent. This, and all other minimizations, were stopped when the rms of the energy gradient elements decreased below 0.05 kcal/(mol Å). Finally, the system was heated gradually, over 0.5 ns from 0 K to 295.15 K at constant volume, using a Langevin thermostat with a collision frequency of 2 ps^−1^. Bonds involving hydrogen atoms were constrained using SHAKE (55), the integration step was 2 fs, slowly varying forces were evaluated every 2 steps using r-RESPA (56, 57), and the maximum distance for the calculation of effective Born radii, as well as the nonbonded interaction cutoff, were set to 64 Å.

For the RXSGLD simulations, we set up 12 replicas. The main one was kept at 295.15 K, whereas the others were running with a generalized Langevin equation (58) with local averaging time for the calculation of the guiding force of 0.2 ps and effective temperatures of 303 K, 312 K, 321 K, 330 K, 339 K, 348 K, 358 K, 368 K, 378 K, 389 K, and 400 K, respectively. In this stage, we did not use r-RESPA, the Langevin collision frequency was 10 ps^−1^, and replica exchanges were attempted every ps. The total duration of the run was 960 ns; of these, we only considered the last 610 ns, to ensure that the system was well equilibrated.

To compute the SWRMSD with respect to a reference frame, we first calculate a score for each residue of each frame. The score is 0 if the secondary structure assigned to that residue in that frame is the same as the one assigned to the same residue in the reference frame, it is 1 if the residue is found as a coil in one of the two frames and in a folded state in the other, and it is 4 if the residue is found in a helix in one of the two frames and in an extended region in the other. Then, we reweigh the RMSD of each frame by multiplying it by the sum of the scores of all residues in that frame. Our specific choice of scores was motivated by a will to give a higher importance to more dramatic differences, such as those between an alpha helix and a beta sheet, whilst not neglecting those between coils and folded regions, which are however much more likely to occur over the whole trajectory, especially at the ends of structured domains.

Relaxation of the full models of AXR3 was carried out in an explicit solvent, using the ff99SB force field (50) with OPC water (59), since this combination is known to give the best results for IDPs (60). The systems were solvated within a rectangular box, leaving a minimum distance of 8 Å between the edges of the box and the solute. Charges were neutralized by the addition of K^+^ and Cl^−^ counterions, in number chosen using the Screening Layer Tally by Container Average Potential (SLTCAP) method (61) to provide an ionic strength of 150 mM. For each model, we carried out an initial minimization of the solvent molecules, constraining the positions of the solute atoms via harmonic restraints of 500 kcal/(mol Å^2^), followed by a minimization of the whole system in the same conditions. Cutoffs were set at 8 Å. The systems were then heated at constant volume over 0.5 ns to 295.15 K using a Bussi thermostat (62). The systems were then let to relax at a constant pressure of 1 bar, with a relaxation time of 1 ps, maintained via a Monte Carlo barostat with volume-change attempts every 100 steps, until all the components of the potential energy were not showing a positive or negative trend over the last 50 ns. The same procedure was followed for the TIR1/auxin-complex structure (12), for which we parametrized the molecule of auxin and the structural cofactor inositol hexakisphosphate using antechamber and the GAFF2 force field (63, 64) with BCC charges (65, 66). The final frames of the two clusters were each separately used to create two more systems including the TIR1/auxin complex. For these, before the final relaxation step, we carried out 2 ns of targeted simulation, over which constraints inferred from the crystal structure were added in steps, as described above. The final frames were then minimized with the same protocol as described above.

The coarse-grained simulations were run using the Southamerican Initiative for a Rapid and Accurate Hamiltonian (SIRAH) force field (67). This choice was motivated by the fact that, unlike other coarse-grained force fields, SIRAH allows for structural changes in folded domains, whose possibility should not be precluded in studying IDPs. For these runs, we built coarse-grained parameters for auxin and inositol hexakisphosphate from those of tryptophan, aspartate, and phosphorylated amino acids, mapped the fine-grained systems to coarse-grained ones, solvated them in rectangular boxes with 20 Å of minimum buffer distance, neutralized charges, and added K^+^ and Cl^−^ counterions up to an ionic strength of 150 mM. The parameter files are included in *SI Appendix*, and they can be used for any other simulation of the system with AMBER and SIRAH simply by passing tir-map.txt as a map file to cgconv and then sourcing the file leaprc-tirauxiaa.txt from within LEaP as part of the preparation of the simulation topology; the other files (aux-lib.txt, aux-frcmod.txt, ip6-frcmod.txt, ip6-lib.txt) are then automatically loaded by LEaP as appropriate. Then, we minimized them with a three-step procedure: First, we minimized only the solvent molecules, with the same protocol as described previously, but with cutoffs of 12 Å; then, we minimized the solvent molecules and the side chains of the proteins together, by imposing lighter harmonic restraints of 2.4 kcal/(mol Å^2^) on the positions of coarse-grained backbone nitrogens and oxygens as well as on auxin and inositol hexakisphosphate; finally, we minimized the whole system without any restraints. Subsequently, we carried out a two-step equilibration: First, we ran 5 ns at constant pressure with restraints of 2.4 kcal/(mol Å^2^) on the positions of all atoms except solvent ones; then, we ran 25 ns with backbone and ligands restraints of 0.24 kcal/(mol Å^2^). Finally, we ran 1 µs of unconstrained production run for each system. In the equilibration steps and production runs, we kept the crystal structure constraints we used before, and the integration step was 20 fs. Native contacts were estimated using a distance cutoff of 12 Å.

All analyses of the trajectories were carried out using cpptraj (68). Structural assignments employed the DSSP method (69). Error bars were calculated using the jackknife method (42), with correlation times estimated via the autocorrelation function of the RMSD of the relevant trajectory or part thereof.

Molecular images and movies were created using UCSF Chimera (70). Solvent-excluded molecular surface visualizations were generated using MSMS (71). Molecular surface images and movies were rendered with PoV-Ray (72).

### Protein Preparation.

The N-terminal half of AXR3 and *axr3-3* were expressed as 6X His-tag (N terminal) fusion proteins in *Escherichia coli* strain Rosetta™ DE3 competent cells (*SI Appendix*, Table S2; Novagen, product code: 70954). These proteins were expressed in minimal media with ^13^C D-glucose and ^15^N ammonium chloride. The maximization of isotope labeling of the expressed protein involved a 125-fold dilution of cell culture in enriched growth media into minimal media with ^13^C D-glucose and ^15^ N ammonium chloride and growth for 16 h (37 °C/200 rpm); followed by a further 40-fold dilution into minimal media for the final period of cell growth and protein expression (induced with 0.5 mM IPTG/18 °C/200 rpm and grown for a further 12 h). The fusion protein was isolated from soluble cell lysate by Co-NTA affinity chromatography and the protein eluted on a gradient of increasing imidazole concentration. Chromatography buffers contained 20 mM sodium phosphate, pH 8.0, 500 mM NaCl, and either 10 mM or 500 mM imidazole for wash and elution buffers, respectively.

For the preparation of unlabeled Arabidopsis TIR1, expression constructs were engineered into the pOET5 transfer vector (Oxford Expression Technologies) to allow coexpression of the fusion proteins His10-eGFP-FLAG-(TEV)-AtTIR1 and His10-(TEV)-AtASK1 (pOET5 AtTIR1 AtASK1) in *Spodoptera frugiperda*9 (Sf9) insect cells and purified with the following modifications. Soluble cell lysate was passed through a HiTrap 1 mL TALON Crude column, followed by a column of ANTI-FLAG® M2 affinity gel (Sigma-Aldrich, product code: A2220) with the sample in a buffer containing 1 mM DTT, 150 mM NaCl, and 10 mM HEPES pH 7.4 and eluted with 100 µg mL^−1^ 3xFLAG peptide (Sigma). Purified TIR1 protein was stored on ice and protein concentrations were assayed by nanodrop (Thermo Fisher Scientific).

### CD Spectroscopy.

The N-terminal half of AXR3 was expressed in *E. coli* BL21 (DE3) using a 2YT medium and then purified using Co-NTA, the same purification strategy as for NMR analyses. Protein was desalted into PNE buffer (sodium phosphate 20 mM pH 6.0, NaCl 150 mM, EDTA 3 mM) and into 20 mM sodium phosphate pH 6.0 using NAP5 columns. CD spectra, in triplicate, were collected on an Applied Photophysics Chirascan spectrometer (software version 4.7.0.194) between 180 and 260 nm using 0.2 mg/mL samples in 2-mm quartz cuvettes. Secondary structure contributions were deconvoluted using CDNN (Applied Photophysics; version 2.10.223).

### NMR Sample Preparation.

All protein samples for NMR analysis were concentrated by ultrafiltration and underwent buffer exchange into 20 mM sodium phosphate pH 6.0, 150 mM NaCl, 3 mM EDTA, 10 mM DTT, cOmplete mini protease inhibitor cocktail (2% v/v; Roche Molecular Biochemicals). Before NMR analysis, D_2_O (5 to 10% v/v depending on the frequency of the spectrometer) was added to the sample.

### NMR Backbone Assignment.

The following NMR experiments were used in the assignment of the backbone of AXR3 DI/DII: HNCA, HNcoCA, HNcaCB, CBcacoNH, HNcaCO, HNCO using ^13^C, ^15^N isotopically labeled protein (290 µM). Parameters are listed in *SI Appendix*, Table S3. All the assignment experiments were performed at 600 MHz at 16.5 °C using an Agilent DDX3 NMR spectrometer with a RT HCN triple resonance probe. The assignment data were analyzed with minimal automation in the software CcpNmr Analysis *SI Appendix*, Table S4.

### Sequential NMR Backbone Assignment through the Prolines in the AXR3 DI/DII Protein.

A set of 2D ^13^CO detected NMR experiments CON, hCAnCO, and hCACO were used in the assignment of prolines in the carbon backbone of AXR3 DI/DII. The parameters for the NMR experiments are described in *SI Appendix*, Table S5. The experiments were performed at 950 MHz at 16.5 °C using a TCI cryoprobe with a cooled amplifier on carbon.

### Identifying and Estimating the Occupancy of the *Cis* and *Trans* Isomer Populations.

The ^13^C_α_ *cis* Pro population was predicted to have an upfield chemical shift of around 0.5 ppm (73, 74). The hCAnCO and hCACO spectra for AXR3 DI/DII show an upfield ^13^C_α_ chemical shift difference of around 0.3 ppm for the *cis* isomer population of P87 compared to the *trans* isomer position. The height and volume of NMR signals assigned to G85 and W86 were determined automatically from the assignment peak list for the ^1^H–^15^N HSQC spectrum within the software CcpNmr Analysis using the peak picking option. The height of the NMR signals was measured by a parabolic method. The NMR experiments were performed at least 12 h after purification.

### ^15^N NOE and ^15^N RDC NMR Experiments of AXR3 DI/DII.

A ^15^ N NOE experiment was performed at 16.5 °C and at 950 MHz. Saturation was achieved with a block of 120 pulses every 5 ms for 4 s and a total recycle delay of 5 s AXR3 DI/DII protein samples were prepared for the RDC experiment, with and without 4.6% peg-hexanol as an alignment medium. The backbone amide (^1^D_NH_) RDC’s were measured using IPAP-HSQC experiments and peak separations were determined with nonlinear lineshape fitting in nmrPipe.

### NMR Analysis of the Auxin Coreceptor Complex.

In our system, the NMR experiments had to be conducted with 5 to 10 µM TIR1 protein at 4 °C and were completed within 18 h from finishing the purification. ^15^N isotopically labeled AXR3 DI/DII protein and unlabeled TIR1 protein was prepared in a 1:3 ratio with 5% D_2_O and measured using a ^1^H–^15^N HSQC experiment following the parameters described in *SI Appendix*, Table S1. The full auxin coreceptor complex was studied by the addition of 200 µM auxin (unlabeled) to the sample. The NMR experiments were initiated with fresh TIR1 and completed within 18 h of finishing the TIR1 purification.

### NMR Shift Back Calculation.

NMR chemical shifts values were back-calculated and averaged from the RXSGLD molecular dynamics simulation structures (n = 600,000) using SHIFTX2 and the python toolset *rc_tools* using snakemake for job management on a cluster of 20 processors.

## Supplementary Material

Appendix 01 (PDF)

Dataset S01 (TXT)

Dataset S02 (TXT)

Dataset S03 (TXT)

Dataset S04 (TXT)

Dataset S05 (TXT)

Dataset S06 (TXT)

Movie S1. Minimum-energy poses of the full AXR3-TIR1 models constructed using Cluster 1. AXR3 is in solid surface representation (pink); TIR1 is in semi-transparent surface/ribbon representation (cyan); also shown are the molecular surfaces of auxin (green) and the structural co-factor inositol hexakisphosphate (InsP6) (red).

Movie S2. Minimum-energy poses of the full AXR3-TIR1 models constructed using Cluster 2. AXR3 is in solid surface representation (pink); TIR1 is in semi-transparent surface/ribbon representation (cyan); also shown are the molecular surfaces of auxin (green) and the structural co-factor inositol hexakisphosphate (InsP6) (red).

## Data Availability

All study data are included in the article, supporting information and/or the Biological Magnetic Resonance Data Bank (accession code BMRB-52109) (https://bmrb.io/) (75).
