# Supplementary material for: Intrinsic disorder and conformational coexistence in auxin coreceptors
Source: Proc Natl Acad Sci U S A. 2023 Sep 27;120(40):e2221286120. doi: 10.1073/pnas.2221286120 (PMC10556615; doi:10.1073/pnas.2221286120)
Supplement: Supplementary file 1 — Appendix 01 (PDF) [file pnas.2221286120.sapp.pdf]

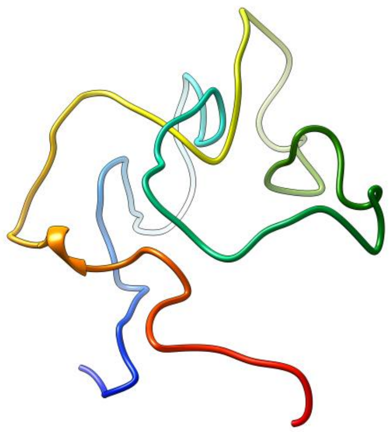

Figure S1. Starting conformation for MD simulations of the N-terminal half of AXR3, obtained via DMPfold. The structure is shown in ribbon representation, with a colouring that passes continuously from pure blue at the N terminal to pure red at the C terminal, for ease of inspection.

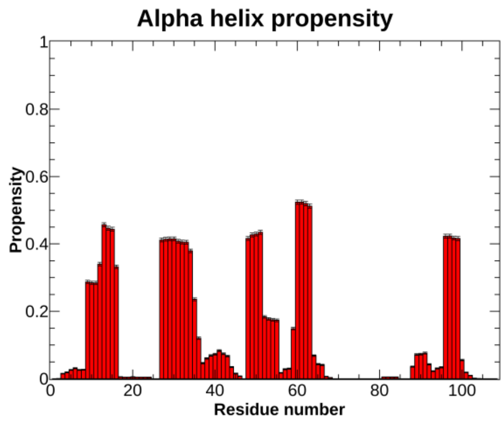

Figure S2. Alpha helix propensities of the N-terminal half of Aux/IAA17.

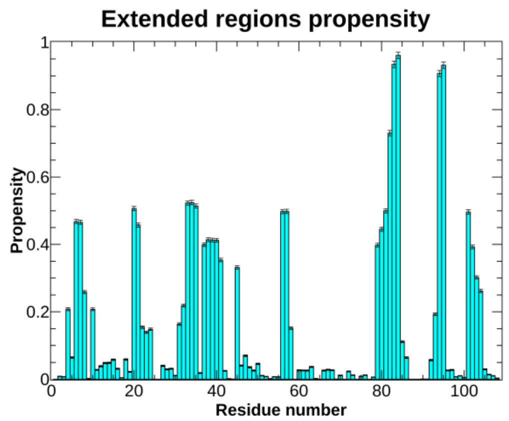

Figure S3. Extended region propensities of the N-terminal half of Aux/IAA17.

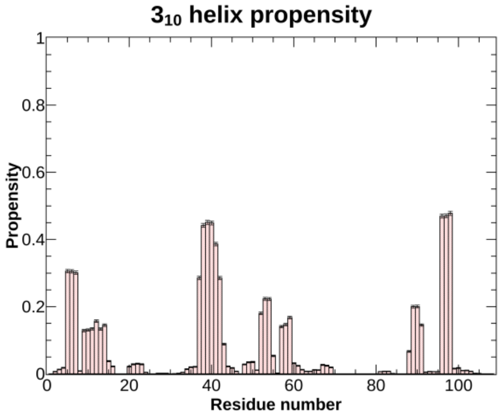

Figure S4. 3_{10} helix propensities of the N-terminal half of Aux/IAA17.

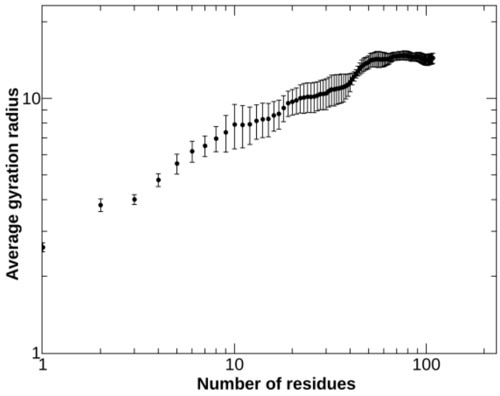

Figure S5. The N-terminal half of Aux/IAA17 does not behave like a pure random coil or self-avoiding walk. The average gyration radius computed over the simulation trajectory for a fragment of the peptide with increasing number of residues does not follow a power-law of the number of residues using, indicating that the first half of the protein does not fall into one of the two classic categories of intrinsically disordered proteins.

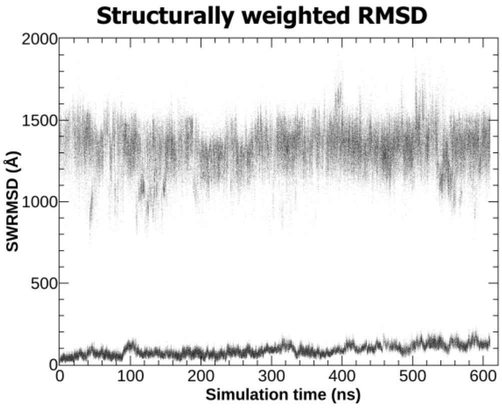

Figure S6. The simulation trajectory of the N-terminal half of Aux/IAA17 contains more than one structural cluster. The SWRMSD of the trajectory separates a first cluster of frames (lower band) from the rest. Repeating the procedure on the remaining frames until the whole trajectory is separated allows us to identify 12 total clusters of structures, of which 2 are the most significant, accounting for more than 90% of total occupancy.

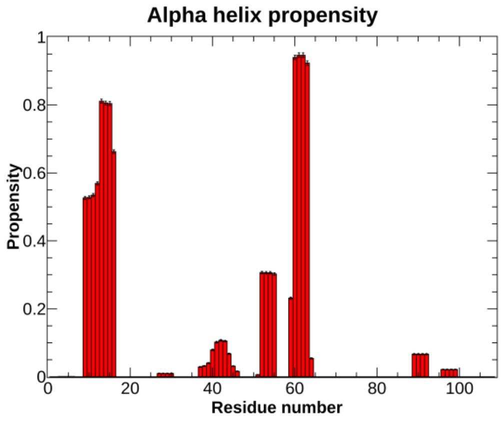

Figure S7. Alpha helix propensities for Cluster 1.

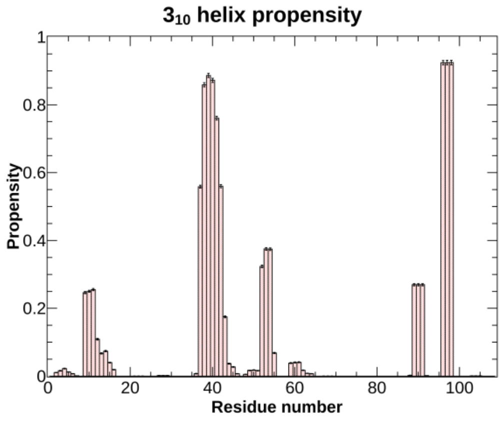

Figure S8. 3_{10} helix propensities for Cluster 1.

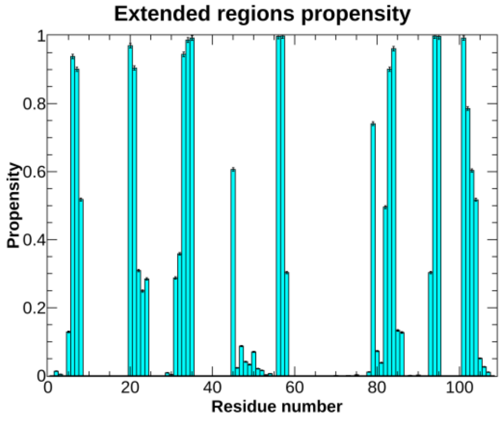

Figure S9. Extended region propensities for Cluster 1.

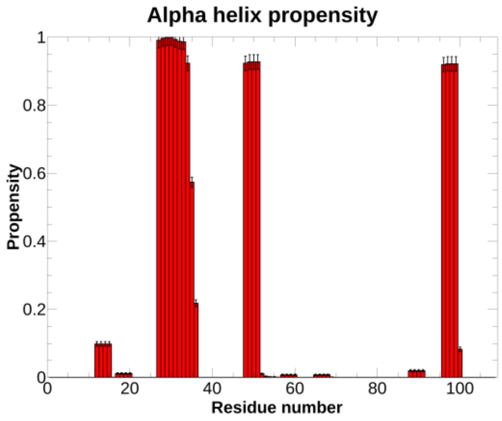

Figure S10. Alpha helix propensities for Cluster 2.

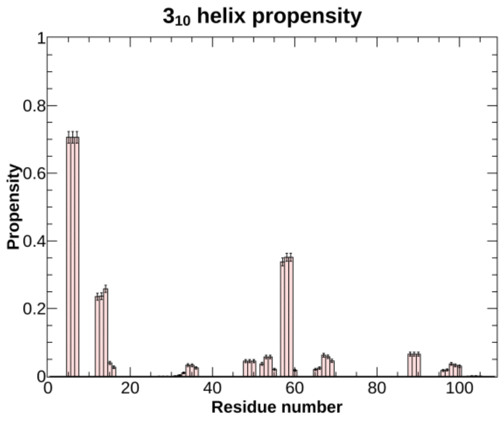

Figure S11. 3_{10} helix propensities for Cluster 2.

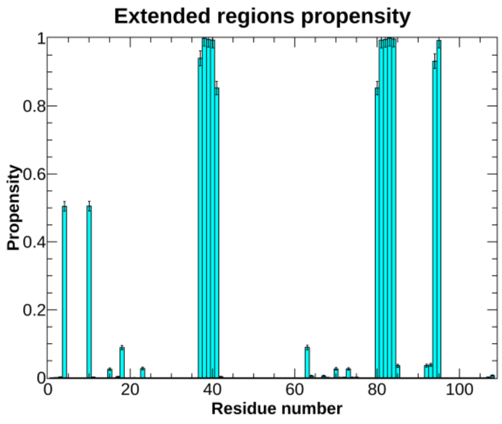

Figure S12. Extended region propensities for Cluster 2.

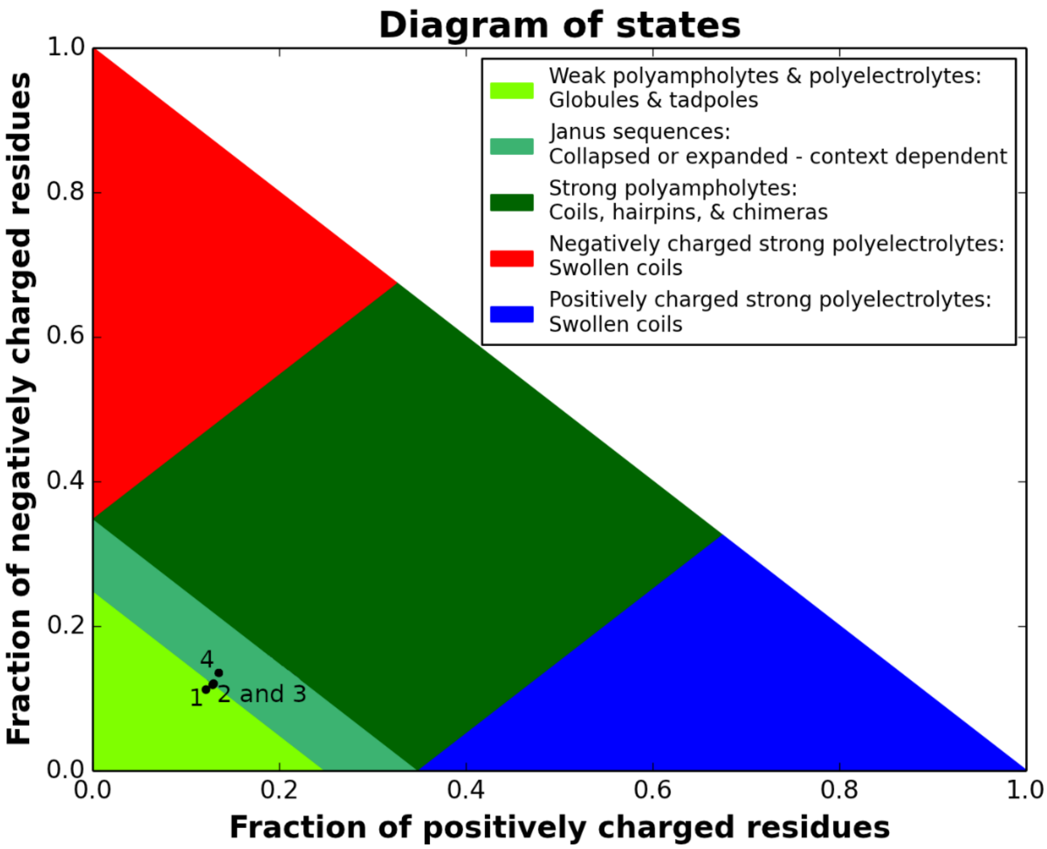

Figure S13. Das-Pappu plot for AXR3. 1) AXR3, which includes the first 101 residues;
2) AXR3_6HN, which includes the first 101 residues, plus a 6-His tag on the N terminus;
3) AXR3_sim, which includes the first 108 residues (which is what we simulated);
4) AXR3_full, which includes the whole sequence of AXR3.

Movie legends

Supplemental Movie 1.

Minimum-energy poses of the full AXR3-TIR1 models constructed using Cluster 1. AXR3 is in solid surface representation (pink); TIR1 is in semi-transparent surface/ribbon representation (cyan); also shown are the molecular surfaces of auxin (green) and the structural co-factor inositol hexakisphosphate (InsP6) (red).

Supplemental Movie 2.

Minimum-energy poses of the full AXR3-TIR1 models constructed using Cluster 2. AXR3 is in solid surface representation (pink); TIR1 is in semi-transparent surface/ribbon representation (cyan); also shown are the molecular surfaces of auxin (green) and the structural co-factor inositol hexakisphosphate (InsP6) (red).

Table S1. Parameters for heteronuclear single-quantum correlation (HSQC) experiment. HSQC experiments to study the auxin co-receptor complex were performed at 4˚C and 950 MHz. All other HSQC experiments were performed at 16.5 ˚C and either 950 or 750 MHz.

**Experiment**	**Recycle Delay (s)**	**Scans**	**Nuclei**		**Spectral width (Hz)**		**Number of complex points**	
			t1	t2	t1	t2	t1	t2
^{1}H-^{15}N HSQC[a]	1	16	^{15}N	^{1}H	2407.2	15243.9	90	1024

a. S. Mori, C. Abeygunawardana, M. O'Neil-Johnson & P.C.M. van Zijl, Improved sensitivity of HSQC spectra of exchanging protons at short interscan delays using a new fast HSQC (FHSQC) detection scheme that avoids water saturation J. Magn. Reson. B 108, 94-98 (1995)

Table S2. Primer list.

**Experiment**	**Primer**	**Sequence**
Cloning AXR3 DI/DII	B1IAA17	5'- GGGGACAAGTTTGTACAAAAAAGCAGGCTGCATGATGGGCAGTGTCGAGCTGAATCT -3'
	IAA17d stopB2	5'- GGGGACCACTTTGTACAAGAAAGCTGGGTATCATTTTTGGCAGGAAACCATCACG -3'
Site-Directed mutagenesis: axr3-3	IAA17SDMF33	5'- TGGCCACCGGGGAGATCATACCGGAAGA -3'
	IAA17SDMR1	5'- TCCCACAACTTGTGCCTTGGCCGGAGGT -3'

Table S3. Parameters for assignment experiments in the analysis of ^{13}C and ^{15}N isotopically labelled AXR3 DI/DII protein. All experiments were performed at 16.5˚C, 600 MHz and with recycling delays of 1 second. Four scans were collected.

Experiment	Nuclei			Spectral width (Hz)			Number of complex data points		
	t1	t2	t3	t1	t2	t3	t1	t2	t3
HNCA[a,b,c]	^{15}N	^{13}C	^{1}H	1600.0	4525.3	6613.8	32	64	1024
HNcoCA[a,b,c]	^{15}N	^{13}C	^{1}H	1600.0	4525.3	6613.8	32	64	1024
HNcaCB[a,b,c]	^{15}N	^{13}C	^{1}H	1600.0	10558.9	6613.8	32	64	1024
CBcacoNH[a,b,c]	^{15}N	^{13}C	^{1}H	1600.0	12067.3	6613.8	32	64	1024
HNcaCO[a,b,c]	^{15}N	^{13}C	^{1}H	1600.0	1600.0	6613.8	48	64	1024
HNCO[a,b,c]	^{15}N	^{13}C	^{1}H	2500.0	1600.0	9615.4	48	64	1024

a. D. R. Muhandiram, L. E. Kay. Gradient enhanced triple resonance three dimensional NMR experiments with improved sensitivity. J. Magn. Res. B. 103, 203-216 (1994).

b. L. E. Kay, G. Y. Xu, T. Yamazaki. Enhanced sensitivity triple resonance spectroscopy with minimal H_2O saturation. J. Magn. Res. A. 109, 129-133 (1994).

c. O. Zhang, L. E. Kay, J. P. Oliver, J. D. Forman-Kay. Backbone ^{1}H and ^{15}N resonance assignments of the N-terminal SH3 domain of drk in folded and unfolded states using enhanced sensitivity pulsed field gradient NMR techniques. J. Biomol. NMR 4, 845-858 (1994).

Table S4. References to software used in the NMR assignment of AXR3 DI/DII protein.

Software	Reference
NMRPipe	F. Delaglio *et al.* NMRPipe: A multidimensional spectral processing system based on UNIX pipes. *J. Biomol. NMR* **6**, 277–293 (1995). https://doi.org/10.1007/BF00197809
CcpNmr Analysis	W. F. Vranken *et al*., The CCPN data model for NMR spectroscopy: Development of a software pipeline. *Proteins: Structure, Function and Genetics* **59**(4), 687–696 (2005).

Table S5. Parameters for proline assignment experiments in the analysis of ^{13}C, ^{15}N isotopically labelled AXR3 DI/DII protein. All experiments were performed at 16.5˚C, 950 MHz with recycling delays of 1.5 seconds and acquisition time of 71.3 ms.

Experiment	Scans	Nuclei		Spectral width (Hz)		Number of complex data points	
		t1	t2	t1	t2	t1	t2
CON[a]	8	^{15}N	^{13}C	3851.5	7183.9	160	512
hCACO[b]	8	^{13}C	^{13}C	7168.5	7183.9	64	512
hCAnCO[b]	16	^{13}C	^{13}C	7168.5	7183.9	180	512

a. W. Bermel, I. Bertini, I. C. Felli, R. Kümmerle, R. Pierattelli. Novel ^{13}C direct detection experiments, including extension to the third dimension, to perform the complete assignment of proteins. J. Magn. Reson. 178(1), 56-64 (2006).

b. W. Bermel, I. Bertini, I. C. Felli, R. Pierattelli. Speeding up ^{13}C direct detection biomolecular NMR spectroscopy. J Am Chem Soc. 131(42):15339-45. (2009).
